# Cost-Effectiveness of Tight Control for Crohn’s Disease With Adalimumab-Based Treatment: Economic Evaluation of the CALM Trial From a Canadian Perspective

**DOI:** 10.1093/jcag/gwac001

**Published:** 2022-03-10

**Authors:** Peter L Lakatos, Gilaad G Kaplan, Brian Bressler, Reena Khanna, Laura Targownik, Jennifer Jones, Yasmine Rahal, Kevin McHugh, Remo Panaccione

**Affiliations:** Department of Medicine, Division of Gastroenterology, McGill University, Montreal, Quebec, Canada; Department of Medicine, Division of Gastroenterology and Hepatology, University of Calgary, Calgary, Alberta, Canada; Department of Medicine, Division of Gastroenterology, University of British Columbia, Vancouver, British Columbia, Canada; London Health Sciences Centre—University Campus, London, Ontario, Canada; Mount Sinai Hospital, Joseph and Wolf Lebovic Health Complex, Toronto, Ontario, Canada; QEII—Victoria Building, Halifax, Nova Scotia, Canada; AbbVie Corporation, Saint-Laurent, Quebec, Canada; AbbVie Corporation, Saint-Laurent, Quebec, Canada; Department of Medicine, Division of Gastroenterology and Hepatology, University of Calgary, Calgary, Alberta, Canada

**Keywords:** Adalimumab, Crohn’s disease, Cost-utility analysis, Tight control

## Abstract

Crohn’s disease (CD) is associated with reduced quality of life, increased absenteeism and high direct medical costs resulting from frequent hospitalizations and surgeries. Tumor necrosis factor–alpha inhibitors (TNFi’s) have transformed the therapeutic landscape and enabled a shift from a symptom control to a treat-to-target strategy. The Effect of Tight Control Management on Crohn’s Disease (CALM) trial demonstrated tight control (TC), with TNFi dose changes informed by biochemical markers of inflammation, achieved higher mucosal healing rates compared with conventional management (CM) based on symptoms. A Markov model compared TC and CM strategies from the perspective of the Canadian public payer using patient-observation data from the CALM trial. A regression model estimated weekly CD Activity Index–based transition matrices over a 5-year horizon and included covariates to improve extrapolation of outcomes beyond the 48-week trial assessment period. Costs of CD-related hospitalizations, biomarker tests and adalimumab injections were sourced from public data. Other direct medical costs, quality-adjusted life-years (QALYs), and incremental cost-effectiveness ratios (ICERs) were calculated. Absenteeism was monetized and included in a sensitivity analysis. Over the 5-year time horizon, TC reduced hospitalization costs by 64% compared with CM. Other direct medical costs were reduced by 22%; adalimumab costs increased by 38%, generating an ICER of $35,168 per QALY gained. Absenteeism costs were reduced by 54%, and, when that was included in the model, TC became dominant compared with CM. Management of CD with TC is cost-effective compared with CM in Canada and is dominant if indirect costs associated with absenteeism are included. Trial registration number: NCT01235689.

## INTRODUCTION

The prevalence and incidence rates of Crohn’s disease (CD) in Canada are among the highest in the world (1,2). This chronic, progressive, lifelong inflammatory disease of the gastrointestinal tract typically manifests in adolescence or early adulthood and results in a substantial disease burden, often affecting patients during critical periods in their education and career establishment (3,4). At the patient level, CD results in a much lower quality of life across all dimensions of health compared with healthy controls, with a significantly elevated risk for premature death (5–7). Suboptimal control of inflammation culminates in complications, including strictures, fistulae, or abscesses that ultimately lead to the need for hospitalization or surgery (8). Conventional treatment algorithms prolong active inflammation by delaying the use of effective therapies (9). As a result, approximately one-third of patients with CD require hospitalization in the first year and roughly half undergo intestinal resection within 10 years of diagnosis (10–12). Accordingly, hospitalization and surgery have historically been the main contributors to the direct medical costs associated with care in CD (13,14). In the last two decades, biologics, and in particular tumor necrosis factor–alpha inhibitors (TNFi’s), have transformed the therapeutic landscape in CD (15,16). Multiple randomized controlled trials have demonstrated the efficacy of biologics in inducing and maintaining clinical remission and, although not consistently observed in real-world analyses (17), reducing the risk for hospitalizations and intestinal resections in patients with moderate to severe CD (17–22). As a result, biologic use has been steadily increasing among patients with CD. Biologic therapy is now responsible for up to two-thirds of the total direct healthcare expenditures in Canada for patients with inflammatory bowel disease (23–25).

Over time, treatment strategies have evolved to include earlier intervention, a shift from symptom control to the use of objective markers of disease control (treat to target) and more intense monitoring of patients (26–28). The justification for earlier use of biologic therapy guided by clinical symptoms and biomarkers is supported by high-quality evidence from the Effect of Tight Control Management on Crohn’s Disease (CALM) trial (29). In this study, higher rates of mucosal healing were achieved in subjects with CD through earlier intervention with a tight control (TC) strategy—adjusting the dosing of biologic therapy in response to the presence of biochemical evidence of inflammation, even in the absence of symptoms—compared with conventional management (CM) based on a symptom-driven strategy. Furthermore, it has been shown that patients who achieved mucosal healing had lower rates of subsequent relapse and hospitalizations (30).

The costs associated with earlier use and more aggressive dose optimization of biologic therapy can be best justified if their use can be shown to be effective in improving meaningful health outcomes, in promoting cost savings later in the course of disease by preventing costly complications, or in reducing disease-related disability. Here we have employed a pharmacoeconomic model to assess the potential cost-effectiveness of broadly adopting the TC strategy outlined in CALM from the perspective of the Canadian public payer.

## METHODS

### Model Structure

**Figure 1.**
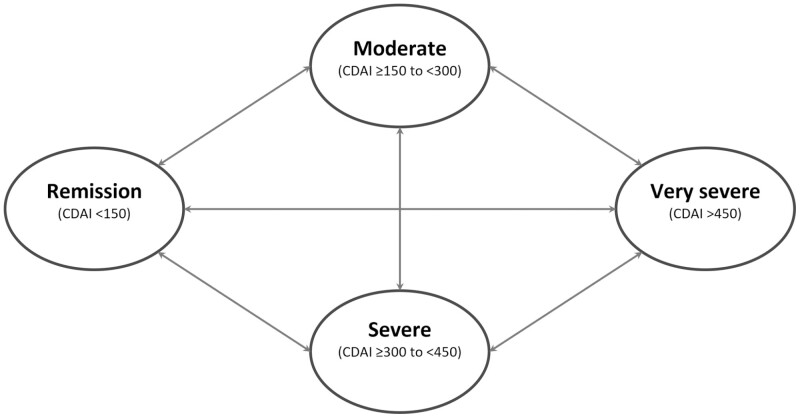
Structure of state transition model. Patients with Crohn’s disease may have different levels of disease activity, included as the CDAI-based health states in the model. Each model week, a patient was predicted to be in one of the health states and could transition from one health state to another based on transition probabilities derived from CALM trial data using a regression model. Hospitalization was included as a toll state. The health states determined patients’ costs, health utility and likelihood of hospitalization.

Details of model design have been published elsewhere (31). In brief, a state transition (or Markov) model was developed to compare TC and CM strategies from the perspective of the Canadian public payer using patient-observation data from the CALM trial (Figure 1) (29,31).

Patients in the model could transition between health states defined by CD Activity Index (CDAI) scores, based on transition probabilities. The health states included in the model were remission (CDAI < 150), moderate (CDAI 150 to <300), severe (CDAI 300 to <450) and very severe (CDAI ≥450), with hospitalization as a toll state. Transition probabilities were estimated using an ordered probit model with a simple regression to assess the treatment effect (TC or CM). Modeling was performed with a 5-year time horizon, and the model included covariates for time since last CDAI measure and a dummy variable that corresponded to the peak clinical remission rates observed in CALM to improve extrapolation of outcomes beyond the 48-week trial assessment period. To allow treatment effects to vary across health states and over time, the model used a specification in which the treatment variable interacted with lagged health states. Predicted health state distributions were linearly interpolated between trial observations to derive weekly health state distributions. In the sensitivity analysis, observed CDAI-based health state distributions were used instead of predicted distributions. Proportion of time in remission was calculated as the number of weeks in remission divided by total modeled weeks.

As described previously, the ordered probit regression indicated a greater likelihood of patients maintaining their health state or moving to a less severe health state with TC versus CM (31).

### Data

Model inputs for baseline characteristics, health state distributions, hospitalization rates conditional on health states and absenteeism were taken from the CALM trial population (Supplementary Table 1) (29). Patients from the CALM trial were assigned health states based on their CDAI scores at each assessment (weeks 0, 2, 6, 11, 23, 35 and 48) using an intent-to-treat approach, and transition probabilities between health states were derived using a regression model (Supplementary Table 2) (31). Missing CDAI values or censored values were imputed using the last-observation-carried-forward approach.

### Direct Medical Costs

Where possible, direct medical costs were sourced or estimated from the provincial public payer perspective to allow an estimation of the variance in cost effectiveness across the Canadian healthcare landscape (Supplementary Table 3), and where appropriate the median cost was used in the base case analysis with the lowest and highest values used in the one-way sensitivity analysis (Table 1). Hospitalization costs for patients with CD sourced from a Manitoba (MB) provincial analysis were used in the base case analysis (14). As CD hospitalization costs were not available for other provinces, data from the Canadian Institute for Health Information (CIHI) Patient Cost Estimator were utilized to generate estimates based on the MB costs, as described in Supplementary Table 4. Other direct medical costs including adalimumab injections, other nonbiologic drugs, C-reactive protein and fecal calprotectin costs were sourced using published literature and provincial reimbursement plans applicable in 2020 (Supplementary Table 3) (32–42). The crude rate of CD-related hospitalizations observed in the CALM trial was reported in events per patient-year for TC and CM (29). In the cost-effectiveness model, inputs of CD-related hospitalization events were estimated from a multivariate probit model (using similar specifications as the ordered probit model for health states). Patients in the TC group received C-reactive protein and fecal calprotectin tests at weeks 0 and 11 and every 12 weeks thereafter. Where needed, cost inputs were converted to 2020 Canadian dollars (43,44).

**Table 1.** Model input values

Model input	Base case	One-way sensitivity analysis
Direct medical costs, $∗		
Hospitalization, per admission	16,491	14, 687; 22,066
Fecal calprotectin test, per test	40.00	N/A
CRP test, per test	10.15	3.72; 16.60
Adalimumab, per 40 mg dose	785.45	N/A
Biosimilar discount rate, %	0	0; 40
Health state distribution, %		
Moderate	74	0; 100
Severe/very severe	26	100; 0
Model time horizon, weeks	260	48, 260
Annual discount rate, %	1.5	0; 3.0
Indirect costs		
Hourly wage, $∗	29.51	24.10; 32.73
Average hours per working week, h	36.9	34.8; 37.2

Data sources for direct and indirect costs are presented in Supplementary Tables 3 and 4. CRP, C-reactive protein.

All costs are in 2020 Canadian dollars.

### Indirect Costs and Costs Associated With Absenteeism

In the CALM trial, patients’ ability to work and perform daily activities in terms of absenteeism due to CD was assessed using the Work Productivity and Activity Impairment (WPAI) questionnaire at weeks 0, 11, 23, 35, and 48 (45). The effect of TC compared with CM was reported as the baseline-corrected percentage change in hours missed out of a standard workweek. Absenteeism was monetized using the Canadian average hourly wage and hours in a standard work week in a non-reference case analysis and the lowest and highest provincial average hourly wage and hours in a standard work week were used for the one-way sensitivity analysis (Table 1 and Supplementary Table 3) (46).

### Quality of Life

The EQ-5D health utility values used in the base case were interpolated from CDAI scores obtained from the CALM trial with algorithms derived using a large dataset from clinical trials in CD (47). Quality of life was measured in the CALM trial using the 36-Item Short-Form Health Survey instrument, administered at the same time as the WPAI, and those results were then transformed to Short-Form 6-Dimension (SF-6D) utilities. These SF-6D scores were not used in the base case analysis as such transformed scores have been shown to have a smaller range and lower variance in values and floor effects; however, the SF-6D estimates were used in the sensitivity analysis (48,49).

### Presentation of Results

Cost-utility outcomes were calculated, including incremental cost-effectiveness ratio (ICER), quality-adjusted life-years (QALYs) monetized at 50,000 Canadian dollars each and cost-effectiveness acceptability curves (CEACs). An annual discount rate of 1.5% was employed for the reference case analysis in accordance with the Canadian Agency for Drugs and Technologies in Health (CADTH) guidance, with non-reference discount rates of 0% and 3% evaluated in the one-way sensitivity analysis (50). One-way sensitivity analyses on key variables were performed to examine how results varied for plausible ranges of selected input variables identified in the literature or through analysis of the CALM trial (Table 1).

A probabilistic sensitivity analysis using 1,000 second-order Monte Carlo simulations was conducted to account for the uncertainty in all model parameters simultaneously, and the Cholesky decomposition of the covariance matrices from the multivariate regressions was used to account for correlated inputs in the probabilistic sensitivity analysis (51).

## RESULTS

In the CALM trial, the risk for CD-related hospitalizations was reduced from 28.0 per 100 patient-years in CM-treated patients to 13.2 per 100 patient-years in TC-treated patients, with regression analysis indicating higher risk for hospitalization for those patients in more severe health states (29,31). Over the 5-year time horizon of the model, this resulted in a 64% reduction in hospitalization costs for patients receiving TC versus CM. Other direct medical costs were also reduced (by 22%) with TC as were absenteeism costs (by 54%) over the 5-year time horizon. Adalimumab costs were higher (by 38%) in the TC group.

In the reference case, which included all direct medical costs, the ICER was $36,051 per QALY gained (Table 2). When absenteeism was included in the analysis, TC was dominant. This indicates that the TC treatment strategy with adalimumab for patients with moderate to severe CD is cost-effective at a willingness-to-pay threshold of $50,000 per QALY gained.

**Table 2.** Results of cost-effectiveness evaluation over 260-week time horizon

	Reference case
Total direct costs, $∗	
TC	133,721
CM	124,835
Difference	8,886
Total costs including absenteeism, $∗	
TC	80,970
CM	90,567
Difference	−9,597
Total QALYs	
TC	3.658
CM	3.412
Difference	0.246
ICER ($ per QALY)	
Direct costs only, $∗	36,051
Including absenteeism	DOMINANT

CM, clinical management; ICER, incremental cost-effectiveness ratio; QALY, quality-adjusted life-years; QC, Quebec; TC, tight control.

All costs are in 2020 Canadian dollars.

**Figure 2.**
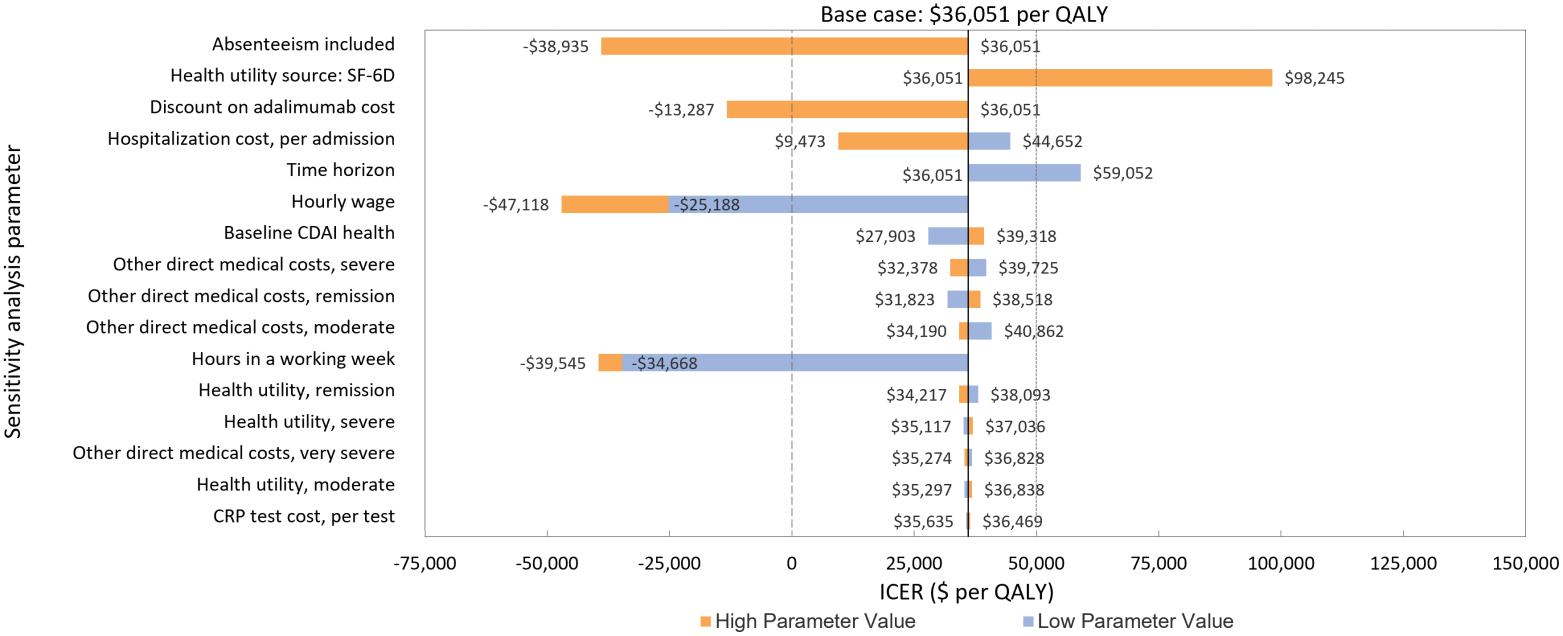
Results from a one-way sensitivity analysis of base-case analysis. Parameters (refer to Table 2 for values) are presented in descending order of model sensitivity. Bars that do not cross the vertical axis represent parameters with one variable only in the sensitivity analysis. Vertical dashed lines represent thresholds for dominance (long dashed lines) and willingness to pay (short dashed lines). SF-6D values derived from the CALM trial were used in the sensitivity analysis. CALM, Effect of Tight Control Management on Crohn’s Disease; CDAI, Crohn’s Disease Activity Index; CRP, C-reactive protein; ICER, incremental cost-effectiveness ratio; QALY, quality-adjusted life-years; SF-6D, Short-Form 6-Dimension. ∗All costs are in 2020 Canadian dollars.

**Figure 3.**
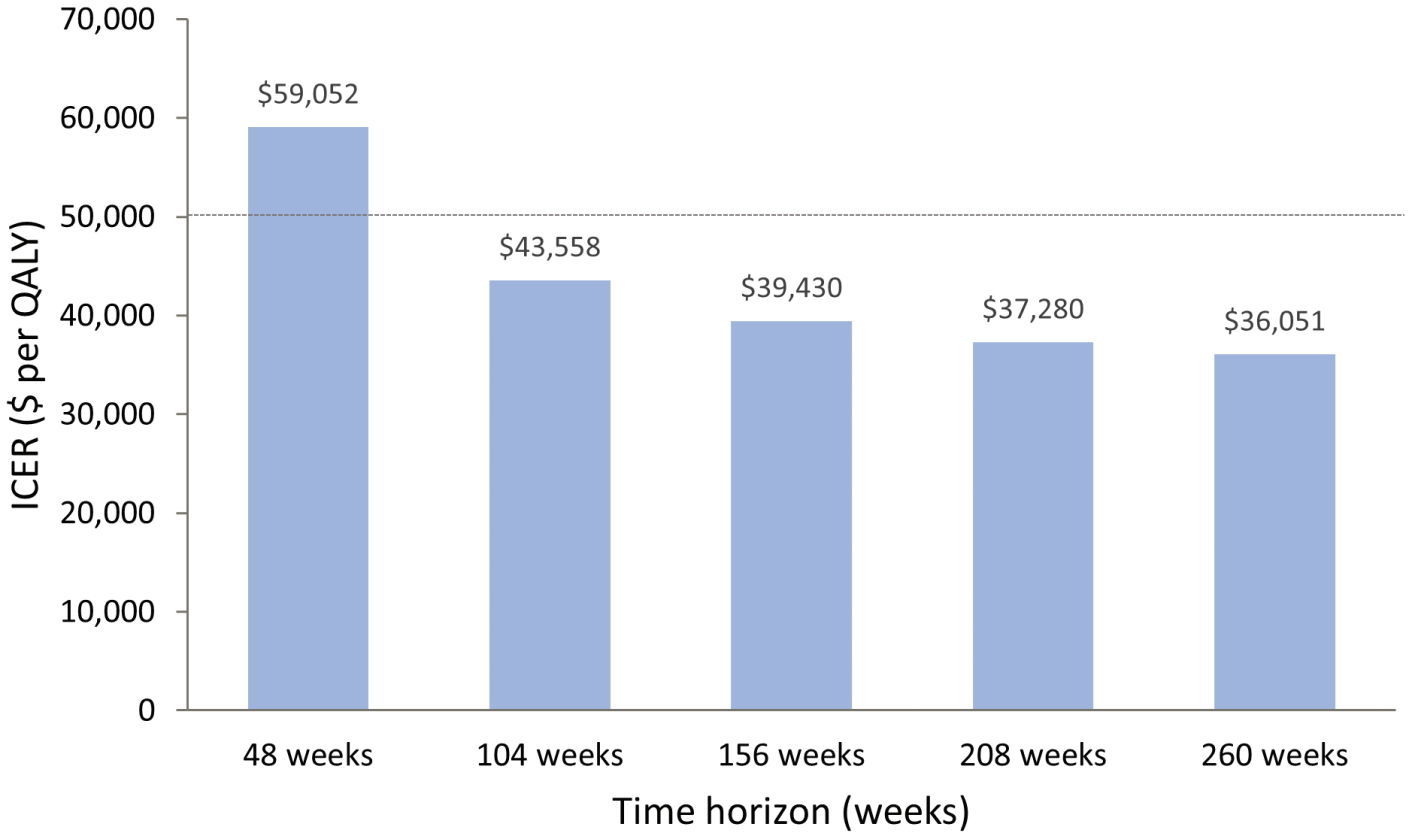
Impact of time horizon on ICER. The ICER was calculated using the base case scenario with only the time horizon changing. Horizontal dashed line represents the threshold for willingness to pay of $50,000∗ per QALY. ICER, incremental cost-effectiveness ratio; QALY, quality-adjusted life-years. ∗All costs are in 2020 Canadian dollars.

**Figure 4.**
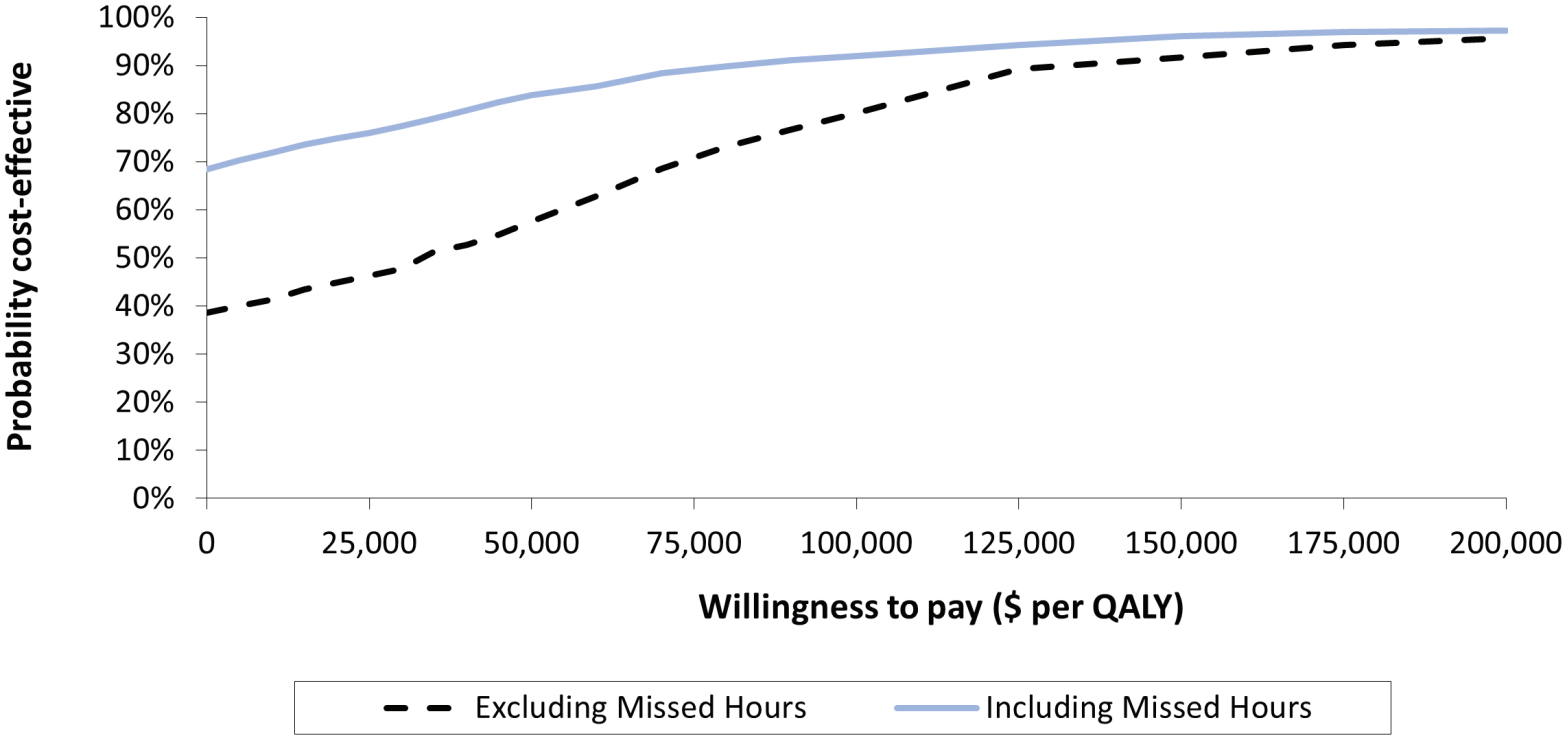
Cost-effectiveness acceptability curves representing the probability of cost-effectiveness of tight control versus clinical management excluding (reference case) and including absenteeism due to Crohn’s disease, at different willingness-to-pay thresholds. Results are depicted for the analysis excluding absenteeism (hashed lines) and including absenteeism (solid line). Results are based on the probabilistic sensitivity analysis, which included 1,000 second-order Monte Carlo simulations in which model variables were simultaneously varied. Vertical line depicts the $50,000 (dashed lines) willingness to pay threshold per QALY gained. ∗All costs are in 2020 Canadian dollars.

The inputs and results of the one-way sensitivity analyses are presented in Table 2 and Figure 2. Model results were most sensitive to the inclusion of absenteeism, the health utility cost source, the cost of adalimumab, and CD-related hospitalization costs. When a time horizon of 48 weeks was used, the ICER was $59,052 per QALY. However, this reduced to $43,558 per QALY if the time horizon was extended to 104 weeks (Figure 3) and continued to decline to the model limit of 260 weeks (base case). Annualized discount rate had a marginal impact on the ICER, ranging from $35,923 to $36,180 per QALY for a discount rate of 0% and 3%. Respectively, the results of the probabilistic sensitivity analysis for the reference case are displayed as a CEAC in Figure 4. The CEAC indicated that 57.6% of simulations were below an ICER of $50,000 per QALY gained in the reference case (excluding absenteeism). When absenteeism was included, 83.8% of simulations were below the same threshold.

## DISCUSSION

This analysis highlights that a TC treatment strategy with adalimumab brings significant clinical and societal benefits for patients with moderate to severe CD in a cost-effective manner. Although the increased use of adalimumab represents a large component of the higher overall direct costs associated with TC, this is partly ameliorated by the benefits of increased remission rates, reduced hospitalizations, and improved quality of life for patients with CD. Furthermore, the resultant savings from reduced workplace absenteeism associated with TC may compensate for the difference in adalimumab expenditure. The model results are also generally robust to sensitivity analyses, indicating that the estimates were reliable, although it should be noted that only approximately 60% of simulations in the probabilistic sensitivity analysis fell below the willingness to pay threshold of $50,000 per QALY.

The CALM trial incorporated economic endpoints and appropriate follow-up time for assessment of economic effects and was not designed to hinder an intent-to-treat analysis. Although an increase in cost to the public payer should be considered, CALM demonstrated that TC results in improved health compared with CM and was considered cost-effective (29). The prevalence of CD in Canada was 368 per 100,000 people in 2018, ranging from as low as 295 per 100,000 in British Columbia to as high as 554 per 100,000 in Nova Scotia (1). For the two most populous provinces in Canada, the prevalence of CD in 2018 was 427 (Quebec) and 335 (Ontario) per 100,000 people (52). However, this must be weighed against the societal benefits of less burden on secondary and tertiary care and improved work productivity.

This study is aligned with current guidance from the CADTH that the analysis be performed from a public payer perspective with a cost-effectiveness threshold of $50,000 and that the effect of time lost from work by patient and caregiver should be included in a nonreference case analysis (50).

The deterministic sensitivity analysis highlighted the model’s sensitivity to inclusion of absenteeism, adalimumab and hospitalization costs, health utility source, the time horizon used, and baseline health state.

The nonreference base case absenteeism cost was calculated using pan-Canadian inputs for the hours worked in a standard work week and the average hourly wage rate for both full- and part-time employees of both sexes aged 15 years or older, with the provincial extremes for both parameters included in the sensitivity analyses. (53).

Adalimumab costs were based on publicly available provincial formulary rates and do not include any negotiated discounts, which may overestimate the true cost to the public payor. When the cost of biosimilar products was included in the model, TC became dominant to CM (54). It should also be noted that, in alignment with CADTH guidance, the model assumes equity in cost and benefit to the public payor, even though in Canada a significant proportion of drug costs (but not hospitalization costs) are assumed by private payors, exacerbating the overestimation of the true cost to the public payor.

Hospitalization costs were derived from a Manitoba-based analysis that included 3,735 people with CD, compared with a population-based group of controls without CD (14). The cost per hospitalization for people with CD was not substantially different from controls, supporting the validity of the analysis, but the incidence of hospitalization (and therefore the overall cost of hospitalizations) was markedly higher for the CD group. As the CIHI patient cost calculator demonstrates, there are significant inter-provincial differences in hospitalization costs. In the absence of primary data for CD-related hospitalization costs outside MB, the CIHI data were used to generate estimates of such costs for other Provinces.

Modeled health states based on CDAI observations were used here, as in previous studies (31), to maximize patient-observation data in the reference case; unmodeled CDAI states were used in the sensitivity analysis. Healthcare resources not measured in the trial, for instance outpatient and emergency department visits, physician consults, radiology and imaging, routine laboratory tests, and use of nonbiologic drugs, were imputed from a UK-based study that stratified resource use by disease severity (55).

The increased ICER obtained using a 48-week period in the sensitivity analysis demonstrated the model’s sensitivity to this parameter. Although the time horizon used in the reference case necessitated predictive modelling of costs associated with adalimumab and hospitalizations, the extension of the time horizon beyond the 48-week assessment period of the CALM trial allows for relevant differences in the future costs and outcomes associated with the TC versus CM treatment strategies to be estimated; an important consideration given the life-long nature of the condition.

Other limitations to this analysis include differences between the CALM study population and real-world experience, which may limit the external validity of the outcomes underpinning this economic model. For example, patients enrolled in CALM were recently diagnosed with CD (median 2.61-month disease duration for both arms), and the results obtained may not be generalizable to a patient population with more established disease histories. The mean age for the CALM cohort was approximately 32 years, which is consistent with a recent diagnosis of CD in Canada (52). However, it does not necessarily reflect the age range of the Canadian CD population as a whole, which includes those living with the condition for many years, an important factor given the increased costs associated with management of CD in the elderly (14).

Operational practicalities may require real-world patient management practices to vary from those employed in CALM, potentially affecting the outcomes achieved in terms of hospitalizations and absenteeism as well as the costs associated with adalimumab use, all of which the model is particularly sensitive to. The analysis focuses on the cost-effectiveness of TC in the CALM population. It does not explore the cost-benefit in specific subgroups (e.g., with severe disease only) nor does it model the spectrum of disease severity or direct and indirect costs in each province. Absenteeism was calculated using a human capital rather than a friction cost approach, which in general leads to an overestimation. However, it may be argued that a human capital approach is most appropriate given the intermittent pattern of absenteeism in patients with CD.

## CONCLUSION

This analysis models the cost-effectiveness of TC versus the conventional symptom-driven treatment strategy in immune suppressant—and biologic-naive patients with CD based on data obtained from a multinational randomized controlled trial. The results demonstrate that, consistent with the UK analysis, TC is cost-effective compared with CM in Canada, strengthening the support for this management strategy from a fiscal as well as a clinical perspective. Furthermore, if indirect costs associated with absenteeism are included, TC is dominant to CM.

## Supplementary Material

gwac001_suppl_Supplementary_Material
