# Supplementary material for: Cost-Effectiveness of Tight Control for Crohn’s Disease With Adalimumab-Based Treatment: Economic Evaluation of the CALM Trial From a Canadian Perspective
Source: J Can Assoc Gastroenterol. 2022 Mar 10;5(4):169–76. doi: 10.1093/jcag/gwac001 (PMC9340647; doi:10.1093/jcag/gwac001)
Supplement: gwac001_suppl_Supplementary_Material [file gwac001_suppl_supplementary_material.docx]

# Cost-Effectiveness of Tight Control for Crohn’s Disease with Adalimumab-based Treatment: Economic Evaluation of the CALM Trial from a Canadian Perspective

## Supplementary Materials

**Table S1.** Baseline characteristics of the subjects enrolled in the CALM trial.

| **Characteristic** | **Tight control  (N=122)** | **Clinical management (N=122)** |
| --- | --- | --- |
| *Demographics* | | |
| Age (years, mean [SD]) | 32.1 (12.0) | 31.1 (11.4) |
| Male, n (%) | 50 (41.0) | 53 (43.4) |
| Disease duration (months, mean [SD]) | 12.7 (27.3) | 10.5 (20.5) |
| *CDAI, n (%)* | | |
| Remission (CDAI <150) | 0 (0.0) | 0 (0.0) |
| Moderate (≥150 CDAI <300) | 89 (73.0) | 92 (75.4) |
| Severe (≥300 CDAI <450) | 33 (27.0) | 29 (23.8) |
| Very severe (CDAI ≥450) | 0 (0.0) | 1 (0.8) |
| *Previous CD-related drug use, n (%)* | | |
| Aminosalycilates | 26 (21.3) | 20 (16.4) |
| Antibiotics | 0 (0.0) | 3 (2.5) |
| Systemic corticosteroids | 6 (4.9) | 7 (5.7) |
| *Employment characteristics* |  |  |
| % work hours missed due to CD, mean (SD) | 35.8 (42.9) | 30.0 (37.4) |

No significant difference (P<0.05) was identified between treatment groups using Wilcoxon rank-sum tests and Chi-square tests to compare categorical and continuous variables, respectively.

**Table S2.** Markov matrices derived from CE model ordered probit specification, based on results from the CALM trial.

| **Transition probabilities up to and including model week 23:** | | | | |
| --- | --- | --- | --- | --- |
| *Tight control* | Remission | Moderate | Severe | Very severe |
| Remission | 0.965 | 0.035 | 0.000 | 0.000 |
| Moderate | 0.300 | 0.657 | 0.043 | 0.000 |
| Severe | 0.009 | 0.434 | 0.544 | 0.014 |
| Very severe | 0.000 | 0.119 | 0.761 | 0.119 |
|  |  |  |  |  |
| *Clinical management* | Remission | Moderate | Severe | Very severe |
| Remission | 0.947 | 0.053 | 0.000 | 0.000 |
| Moderate | 0.253 | 0.689 | 0.057 | 0.000 |
| Severe | 0.003 | 0.308 | 0.658 | 0.031 |
| Very severe | 0.000 | 0.011 | 0.515 | 0.474 |
|  |  |  |  |  |
| **Transition probabilities after model week 23:** | | | | |
| *Tight control* | Remission | Moderate | Severe | Very severe |
| Remission | 0.934 | 0.065 | 0.000 | 0.000 |
| Moderate | 0.204 | 0.717 | 0.079 | 0.000 |
| Severe | 0.004 | 0.323 | 0.645 | 0.028 |
| Very severe | 0.000 | 0.069 | 0.739 | 0.191 |
|  |  |  |  |  |
| *Clinical management* | Remission | Moderate | Severe | Very severe |
| Remission | 0.906 | 0.094 | 0.000 | 0.000 |
| Moderate | 0.166 | 0.732 | 0.102 | 0.000 |
| Severe | 0.001 | 0.211 | 0.728 | 0.060 |
| Very severe | 0.000 | 0.005 | 0.400 | 0.595 |

**Table S3.** Canadian and provincial model inputs

| **Model input** | **Canada** | **BC** | **AB** | **SK** | **MB** | **ON** | **QC** | **NB** | **PEI** | **NS** | **NL** |
| --- | --- | --- | --- | --- | --- | --- | --- | --- | --- | --- | --- |
| Hospitalization costs ($ per admission)^1^ |  |  |  |  | 12,940 |  |  |  |  |  |  |
| Conversion factor from MB cost (See Table S4) | 1.058 | 1.043 | 1.338 | 1.080 | 1 | 0.969 | 1.103 | 0.891 | 0.952 | 1.010 | 0.909 |
| 2020 CAD equivalent^2^ | 17,444 | 17,205 | *22,066* | 17,803 | **16,491** | 15,979 | 18,189 | *14,687* | 15,707 | 16,662 | 14,997 |
| Fecal Calprotectin test ($ per test)^3^ |  | 40 |  |  |  |  |  |  |  |  |  |
| 2020 CAD equivalent^2^ |  | **41.99** |  |  |  |  |  |  |  |  |  |
| CRP test, 2020 ($ per test)^4-7^ |  | 10.31 | **10.15** | *16.60* | 8.75 | *3.72* |  |  |  |  |  |
| Adalimumab cost, 2020 ($ per 40mg dose) ^8-17^ |  | 824.72 | 785.45 | **785.45** |  | 785.45 | 714.24 | 784.45 |  |  | 784.45 |
| Biosimilar discount^8-17^ |  | 40% | 40% | 40% |  | *40%* | 33% | 40% |  |  | 40% |
| Hourly wage, 2020 ($)^18^ | **29.51** | 29.76 | *32.73* | 29.13 | 26.32 | 30.24 | 28.2 | 24.46 | *24.10* | 25.31 | 27.35 |
| Average hours per working week, 2020 (h)^19^ | **36.9** | *34.8* | 36.4 | 37.1 | 36.1 | 35.9 | 35.3 | 36.7 | 37.0 | 35.7 | *37.2* |

Blank cells: direct costs not publicly available. Bolded values are those used for the base case scenario; those in italics are used for the one-way sensitivity analysis.

**Table S4.** Estimation of provincial variance in Crohn’s disease hospitalization costs

| **CIHI case mix groups ($ per admission)** | **Canada** | **BC** | **AB** | **SK** | **MB** | **ON** | **QC** | **NB** | **PEI** | **NS** | **NL** |
| --- | --- | --- | --- | --- | --- | --- | --- | --- | --- | --- | --- |
| Inflammatory bowel disease | $6,290 | $6,423 | $8,294 | $6,420 | $5,986 | $5,836 | $6,043 | $5,331 | $6,343 | $6,048 | $5,373 |
| [Province/MB] Ratio | 1.051 | 1.073 | 1.386 | 1.073 | 1 | 0.975 | 1.010 | **0.891** | 1.060 | **1.010** | 0.898 |
| Colostomy/enterostomy | $23,055 | $22,660 | $29,061 | $23,447 | $21,719 | $21,045 | $23,955 | $18,631 | $20,687 | $22,332 | $19,751 |
| [Province/MB] Ratio | 1.062 | **1.043** | **1.338** | **1.080** | 1 | **0.969** | **1.103** | 0.858 | **0.952** | 1.028 | **0.909** |
| Repair/fixation & other moderate intervention on lower GI tract | $6,730 | $6,572 | $8,254 | $7,527 | $6,673 | $6,026 | $6,962 | $5,352 | $5,945 | $5,999 | $6,384 |
| [Province/MB] Ratio | 1.009 | 0.985 | 1.237 | 1.128 | 1 | 0.903 | 1.043 | 0.802 | 0.891 | 0.899 | 0.957 |
| Other GI disorder | $4,761 | $4,784 | $5,735 | $4,572 | $4,082 | $4,427 | $4,880 | $4,286 | $4,121 | $3,886 | $4,080 |
| [Province/MB] Ratio | 1.166 | 1.172 | 1.405 | 1.120 | 1 | 1.085 | 1.195 | 1.050 | 1.010 | 0.952 | 1.000 |
| General symptom/sign | $6,061 | $5,853 | $7,268 | $5,488 | $5,730 | $5,426 | $6,681 | $5,301 | $5,344 | $5,921 | $4,377 |
| [Province/MB] Ratio | **1.058** | 1.021 | 1.268 | 0.958 | 1 | 0.947 | 1.166 | 0.925 | 0.933 | 1.033 | 0.764 |

Estimated average costs for hospitalization of patients aged ≥18 years were sourced from the Canadian Institute for Health Information (CIHI) patient cost calculator, for the case mix groups listed above.^20^ All case mix groups were from the Digestive System major clinical category, except ‘General symptom/sign’ which was included for balance. Hospitalization costs were then normalized to Manitoba costs (the reference case) and the median ratio (in bold) from the selected case mix groups was used in Table S3 to transform the MB CD hospitalization cost to a comparable estimate for each province.

1. Bernstein CN, Longobardi T, Finlayson G, Blanchard JF. Direct medical cost of managing IBD patients: a Canadian population-based study. Inflamm Bowel Dis 2012;18(8):1498-508. DOI: 10.1002/ibd.21878.

2. Inflation calculator. Bank of Canada. (<https://www.bankofcanada.ca/rates/related/inflation-calculator/>).

3. Zhang W, Wong CH, Chavannes M, Mohammadi T, Rosenfeld G. Cost-effectiveness of faecal calprotectin used in primary care in the diagnosis of inflammatory bowel disease. BMJ Open 2019;9(4):e027043. DOI: 10.1136/bmjopen-2018-027043.

4. Schedule of fees for the laboratory services outpatient. British Columbia Provincial Health Services Authority. (<http://www.bccss.org/clinical-services/bcaplm/health-professionals/outpatient-payment-schedule>).

5. Medical procedure list. Alberta Health Services. (<https://open.alberta.ca/dataset/30add047-29c2-4fc7-83b5-a8ab78605cdd/resource/8a42e270-fa32-4959-9070-e93cc0c1fa21/download/health-somb-medical-procedure-list-2020-03.pdf>).

6. Payment schedule for insured services provided by a physician. Saskatchewan Ministry of Health, Medical Services Branch. (<https://www.ehealthsask.ca/services/resources/establish-operate-practice/Documents/Payment-Schedule-April-1-2021.pdf>).

7. Schedule of benefits for laboratory services. Ontario Health Insurance Plan. Laboratory and Genetics Branch. (<https://health.gov.on.ca/en/pro/programs/ohip/sob/lab/lab_mn2020.pdf#:~:text=The%20Schedule%20of%20Benefits%20for%20Laboratory%20Services%28Schedule%29%20provides,eligibility%20criteria%20to%20claim%20fees%20for%20those%20services>.).

8. BC Pharmacare Formulary Search. BC Pharmacare. (<https://pharmacareformularysearch.gov.bc.ca/>).

9. Alberta Drug Benefit List. Alberta Blue Cross. (<https://idbl.ab.bluecross.ca/idbl/load.do>).

10. Saskatchewan online formulary database. Government of Saskatchewan, Ministry of Health. (<https://formulary.drugplan.ehealthsask.ca/SearchFormulary/BG/705299>).

11. Manitoba Drug Benefits and Interchangeability Formulary. (<https://www.gov.mb.ca/health/mdbif/index.html>).

12. Drugs Funded by Ontario Drug Benefit (ODB) Program. Ministry of Health and Long-Term Care. (<http://www.health.gov.on.ca/en/pro/programs/drugs/odbf_eformulary.aspx>).

13. List of medications. Bibliothèque et Archives nationales du Québec. (<https://www.ramq.gouv.qc.ca/sites/default/files/documents/liste-med-2021-08-18-en.pdf>).

14. New Brunswick drug plans formulary. Government of New Brunswick. (<https://www2.gnb.ca/content/gnb/en/departments/health/MedicarePrescriptionDrugPlan/NBDrugPlan/ForHealthCareProfessionals/NewBrunswickDrugPlansFormulary.html>).

15. PEI Pharmacare Formulary. Health PEI. (<https://www.princeedwardisland.ca/en/information/health-pei/pei-pharmacare-formulary>).

16. Nova Scotia Pharmacare. Department of Health and Wellness. (<https://novascotia.ca/dhw/pharmacare/formulary.asp>).

17. NLPDP Coverage Status Table. Newfoundland and Labrador Prescription Drug Program. (<https://www.health.gov.nl.ca/health/prescription/coverage_status_table.pdf>).

18. Employee wages by industry, annual. Statistics Canada. (<https://www150.statcan.gc.ca/t1/tbl1/en/tv.action?pid=1410006401&pickMembers%5B0%5D=1.11&pickMembers%5B1%5D=2.2&pickMembers%5B2%5D=3.1&pickMembers%5B3%5D=5.1&pickMembers%5B4%5D=6.1&cubeTimeFrame.startYear=2020&cubeTimeFrame.endYear=2020&referencePeriods=20200101%2C20200101>).

19. Average usual and actual hours worked in a reference week by type of work (full- and part-time), annual. Statistics Canada. (<https://www150.statcan.gc.ca/t1/tbl1/en/tv.action?pid=1410004301>).

20. Patient cost estimator. Canadian Institute for Health Information. (<https://www.cihi.ca/en/patient-cost-estimator>).
